# Phase angle as a key marker of muscular and bone quality in community-dwelling independent older adults: A cross-sectional exploratory pilot study

**DOI:** 10.1016/j.heliyon.2023.e17593

**Published:** 2023-06-23

**Authors:** Alexandre Duarte Martins, João Paulo Brito, Nuno Batalha, Rafael Oliveira, Jose A. Parraca, Orlando Fernandes

**Affiliations:** a Comprehensive Health Research Centre (CHRC), Departamento de Desporto e Saúde, Escola de Saúde e Desenvolvimento Humano, Universidade de Évora, Largo Dos Colegiais, 7000-727, Évora, Portugal; b Life Quality Research Centre, 2040-413, Rio Maior, Portugal; c Sports Science School of Rio Maior, Polytechnic Institute of Santarém, 2040-413, Rio Maior, Portugal; d Research Centre in Sport Sciences, Health Sciences and Human Development, 5001-801, Vila Real, Portugal

**Keywords:** Aging, Elderly, Bioelectrical impedance analysis, Bone mineral density, Functional capacity, Skeleton

## Abstract

The aim of the present cross-sectional exploratory pilot study was to analyze the ability of the Phase Angle (PhA) to predict physical function, muscle strength and bone indicators, upon adjusting for potential confounders [age, sex, lean mass, and body mass index (BMI)]. This study included 56 physically independent older adults (age, 68.29 ± 3.01 years; BMI, 28.09 ± 4.37 kg/m^2^). A multi-frequency segmental bioelectrical impedance analysis was used to measure PhA at 50 KHz. Additionally, physical function was assessed through four functional capacity tests [30-sec chair‐stand; seated medicine ball throw (SMBT); timed up & go; and 6-min walking test (6 MWT)], muscle strength through the handgrip test (dominant side) and maximal isokinetic strength of the dominant knee flexor and extensor. Moreover, bone indicators and body composition were assessed through the dual energy X-ray absorptiometry.

PhA was significantly associated with SMBT (r = 0.375, effect size (ES) = moderate); 6 MWT (r = 0.396, ES = moderate); 30-sec chair‐stand (rho = 0.314, ES = moderate); knee extension (rho = 0.566, ES = large) and flexion (r = 0.459, ES = moderate); handgrip (rho = 0.432, ES = moderate); whole-body bone mineral content (BMC) (r = 0.316, ES = moderate); femoral neck BMC (r = 0.469, ES = moderate); and femoral neck bone mineral density (BMD) (rho = 0.433, ES = moderate). Additionally, the results of multiple regression analysis demonstrated that PhA is significantly associated with SMBT (*p* < 0.001; R^2^ = 0.629), 6 MWT (*p* = 0.004; R^2^ = 0.214), knee extension (*p* < 0.001; R^2^ = 0.697), knee flexion (*p* < 0.001; R^2^ = 0.355), handgrip test (*p* < 0.001; R^2^ = 0.774), whole-body BMC (*p* < 0.001; R^2^ = 0.524), femoral neck BMC (*p* = 0.001; R^2^ = 0.249), and femoral neck BMD (*p* = 0.020; R^2^ = 0.153). The results of the preliminary analysis suggested that PhA is linked to muscle strength and some factors related to physical function and bone quality in community-dwelling older adults.

## Highlights

• Phase angle is a key indicator of health in older adults.
• Phase angle can predict physical function and muscle strength.
• Phase angle can be used as a marker of bone quality.

## 1 Introduction

Aging is a process involving several quantitative and qualitative changes in skeletal muscle structure, function, and in bone indicators, which increase the risk of fractures, contributing for independence loss and autonomy. Muscle function is becoming increasingly vital in aging due to increased life expectancy [1]. A weak muscle function may be a symptom of sarcopenia condition, which is a risk factor for several adverse health outcomes, including death [2]. Likewise, another worldwide public health concern is osteoporosis which is characterized by low bone mass and microarchitectural changes in the bone tissue [3]. Osteoporosis is considered a risk factor for fractures in the elderly population. Accordingly, there is a growing need to detect several adverse outcomes related to muscle and physical function and bone indicators.

According to the *European Working Group on Sarcopenia in Older People 2* (EWGSOP2), physicians and clinicians should appeal to the imaging methods, *i.e.*, magnetic resonance imaging and computed tomography, or/and dual-energy X-ray absorptiometry (DXA), or/and bioelectrical impedance analysis (BIA) to assess muscle quantity or quality [2]. Also, whole-body DXA has been described as the gold standard for the measurement of bone mineral density (BMD) and bone mineral content (BMC) at the preferential regions of osteoporotic fractures (hip, spine, and distal forearm) in order to diagnose osteoporosis and other bone diseases [4]. Indeed, there is a relationship between low levels of muscle mass and the number of fractures [5], and it has been shown that it is essential to have good levels of lean and fat mass for having good levels of BMD [6]. However, due to the high equipment costs, lack of portability, and patients’ exposure to X-rays, DXA measurement is not commonly used in primary health care.

Alternatively, physicians and clinicians may resort to the BIA-derived phase angle (PhA) measurements to assess muscle quality [[7], [8], [9], [10]]. It is important to consider its usefulness in physiological changes such as fragility associated with aging and adaptations to changes in physical activity. In inflammatory processes involving oxidative stress and changes in cell membranes, PhA can be a critical factor in distinguishing the degree of inflammation and cell oxidation within a morphofunctional evaluation protocol [11,12]. Hence, PhA from BIA has been described as a global health marker [13] and as a muscle index [14] that can predict several outcomes in older adults, such as physical and muscle function [[15], [16], [17], [18]], muscle mass [19], inflammatory and oxidative stress biomarkers [20,21], total and regional BMD [22], mortality risk [23,24], upper body strength, agility, and dynamic balance, regardless of the potential confounding effects of sex, age, and skeletal muscle [16]. In line with the previous statements, it was also possible to verify connections between PhA and the 30-seconds (sec) chair‐stand test, timed up & go (TUG) test, arm curl test and 6-min walking test (6 MWT) in older women [25]. Despite these results, several outcomes have been identified as influencers of PhA, including age, sex, lean mass and body mass index (BMI) [26]. In this sense, several studies have used these outcomes as potential confounders in order to remove the confounding factor for the interpretation of PhA values [16,20,27,28].

Even though there are promising results, the use of the BIA is subject to limitations. Therefore, its validation with other techniques in clinical use through morphofunctional assessment is probably one of the most important aspects when it comes to the external validation of the results of PhA. In this sense, the support of other morphological techniques and the integration of data in the assessment of functionality, such as handgrip strength and functional tests is important [12].

Despite the findings mentioned before, there is a gap in the literature regarding the relationship between PhA and some indicators from gold standard methods, namely isokinetic assessment and DXA measurement of bone indicators [22]. To the best of our knowledge, no other research was found to investigate the relationship between PhA and isokinetic parameters, such as peak torque. Some studies considered that this parameter declines faster than muscle strength [29,30] and, consequently, it can be considered a more important predictor of physical function [31,32].

Regarding muscle power, Rodríguez-Rosell et al. [33] referred that this measurement could be challenging for older adults, since it requires high technical skills, sufficient balance and coordination, proper equipment and familiarization, as well as multiple attempts. In a recent study with future lines of research on phase angle [12], the authors advised that PhA is inversely related to muscle mass and strength in older adults and may be considered a good bioelectrical marker to identify patients at risk of sarcopenia [27], and suggest that researchers investigate whether PhA has a good predictive capacity in relation to the diagnostic components of sarcopenia, such as muscle power and physical function. Falling is not unusual among community-dwelling older adults, especially the elderly, and lower muscle strength is an important issue to address in order to prevent falls. It seems that subjects with higher PhA displayed greater muscle power of the lower limbs [34]. The importance of muscle power lies in its relationship with the increasing incidence of falls [35,36].

Therefore, the present cross-sectional exploratory pilot study investigated the relationship between PhA with body composition parameters, physical function, muscle strength, and bone indicators in community-dwelling independent older adults. We established associations between the PhA and the parameters that characterize the functionality of the elderly. Lastly, this study also analysed the ability of the PhA to predict body composition parameters, physical function, muscle strength, and bone indicators, after adjusting for potential confounders (*i.e.*, age, sex, lean mass, and BMI). The study hypothesis is that PhA can predict, and it is associated with muscle strength and some factors related to physical function and bone quality.

## 2 Materials and methods

### 2.1 Design and participants

**Fig. 1:**
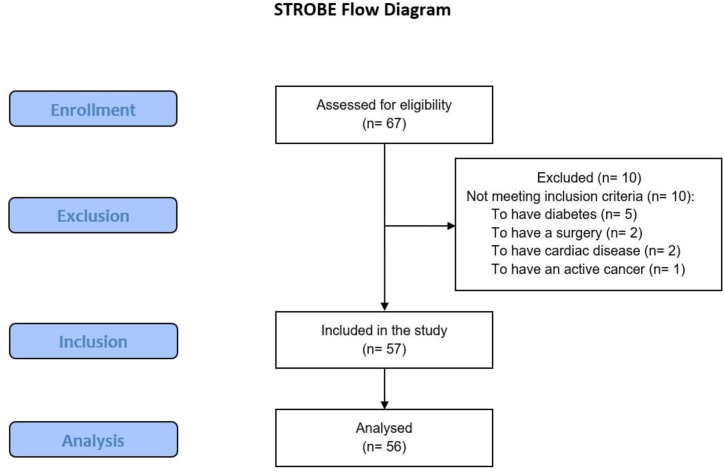
Study enrolment STROBE flow chart.

The present cross-sectional exploratory pilot study assessed the relationship between PhA with body composition parameters, physical function, muscle strength, and bone indicators in independent older adults and it complied with the STROBE Statements (Strengthening the Reporting of Observational Studies in Epidemiology). Sixty-seven participants volunteered for the free tests advertised in local media, invitations sent to daycares, health centers, and associations of older adults in the middle-south areas of Portugal between January and April 2022 were studied. After the selection process (Fig. 1), this study enrolled a total of 56 older adults [mean ± standard deviation (SD); age, 68.29 ± 3.01 years; height, 158.38 ± 6.89 cm; weight, 70.43 ± 11.71 kg; fat mass, 26.87 ± 6.47 kg; and body mass index (BMI), 28.09 ± 4.37 kg/m^2^]. Inclusion criteria were: (i) being at least 65 years old; (ii) able to walk independently; and (iii) perform the tasks of daily living. The exclusion criteria were: (i) having diabetes or/and cardiac diseases; (ii) being submitted to surgery in the last 6 months, and (iii) having active oncology disease.

### 2.2 Sample size and ethics approval

An a priori sample size calculation was performed on G-power software [37] for linear multiple regression (α level = 0.05 and number of predictors = 4), with the result showing that 53 participants are required to achieve a sample size power of 95.2%. Moreover, this research was approved by the ethics committee of the seeding institution (approval no. 22030), and it was implemented in accordance with the World Medical Association's Declaration of Helsinki for human studies. All volunteers were informed about the study's aims and potential benefits and risks and gave their written informed consent to be enrolled in the study.

### 2.3 Procedures

All assessments were performed in two days with an ambient temperature and relative humidity of 22–23 °C and 50–60%, during the first and second day respectively. On the first day, anthropometric, PhA, and DXA assessments were performed in the morning period from 08:30 a.m. to 10:30 a.m., since all participants were instructed to be in a fasted state with empty bladder and without doing exercise, drinking alcohol and coffee in the previous 24 h.

On the second day, participants completed the muscle strength and physical function assessments in the morning period from 09:00 a.m. to 01:00 p.m., having been instructed to bring comfortable clothing. All measurements were conducted by the same researcher to minimize possible errors and the order of the measurements was the same for all participants [warm-up, 30-sec chair‐stand test, TUG test, seated medicine ball throw (SMBT), handgrip strength, 6 MWT, and maximal unilateral isokinetic strength].

### 2.4 Variables and instruments

#### 2.4.1 Anthropometric and phase angle assessment

Weight and height measurements were collected with participants wearing light clothes and no shoes, standing in the Frankfurt horizontal position through an electronic scale (TANITA®, MC 780 MA, Amsterdam, Netherlands) and stadiometer (SECA® 220, Hamburg, Germany) to the nearest 0.01 kg (kg) and 0.1 cm (cm), respectively. Afterwards, the BMI values were assessed using the standard formula: BMI = body mass (kg)/height^2^ (m^2^). Table 1 presents the results of BMI in accordance with the thresholds defined by World Health Organization classification.

**Table 1:** Demographic and general characteristics of the participants.

Characteristics	Men (N = 14)	Women (N = 42)	Total (N = 56)	*p*-Value
Age (years)^a^	70.57 ± 3.79	67.52 ± 2.28	68.29 ± 3.01	0.001^c^
Height (cm)^a^	165.82 ± 6.85	155.89 ± 4.86	158.38 ± 6.89	0.001^c^
SBP (mmHg)^a^	135.64 ± 9.16	131.43 ± 13.12	132.48 ± 12.31	0.271
DBP (mmHg)^a^	78.50 ± 8.68	80.14 ± 8.39	79.73 ± 8.42	0.532
Phase Angle (°)^a^	5.83 ± 0.53	5.42 ± 0.45	5.52 ± 0.49	0.007^c^
BMI (kg/m^2^)^b^				0.010^c^
Underweight (18.5)	0 (0)	0 (0)	0 (0)	
Normal weight (18.5–24.9)	4 (29)	13 (31)	17 (30)	
Overweight (25–29.9)	8 (57)	14 (33)	22 (39)	
Obese (30–34.9)	2 (14)	10 (24)	12 (21)	
Extremely Obese (>35)	0 (0)	5 (12)	5 (10)	

Abbreviations: N, number; cm, centimetres; Kg, kilograms; SD, standard deviation; mmHg, millimeter of mercury; SBP, systolic blood pressure; DBP, diastolic blood pressure.

a Data presented in mean and standard deviation.

b Data presented in frequencies and percent.

c Differences between males and females (*p* ≤ 0.05).

PhA value measured in degrees (°) was assessed using a multifrequency tetrapolar instrument (InBody® S10, Model JMW140, Biospace Co, Ltd., Seoul, Korea) at 50 kHz (Khz) based on a previous study [38]. Before the beginning of the assessment, all metallic objects were removed. Subsequently, the contact points where the electrodes were placed on the skin were cleaned with ethyl alcohol and hydrophilic cotton, and the participants remained in the supine position, still and quiet for 10 min. Finally, after this rest period and in according to the manufacturer instructions to assess the multi-segmental frequency analysis, eight electrodes were placed in the following tactile points: thumbs and middle fingers of both hands, and the foot electrodes were positioned between participant's anklebone and heel.

#### 2.4.2 Dual energy x-ray absorptiometry assessment

The participant's body composition was determined through the following variables: (i) fat mass (FM) in kg; (ii) percentage of FM (%FM); and (iii) lean mass in kg. While for bone mineral values, the BMC in grams (g) and BMD in g/cm^2^ were measured. All variables were assessed by DXA (DXA, Hologic QDR, Hologic, Inc., Bedford, MA, USA). Whole-body and dominant femoral neck were measured according to standard operating procedures [39].

Prior to scanning, all participants were placed in the standard position, with subjects lying supine, without jewellery or metal buttons, with light clothing, and barefoot. Scans were performed with the subjects lying in the supine position along the table's longitudinal centerline axis. Feet were taped together at the toes to immobilize the legs while the hands were maintained in a pronated position within the scanning region. Participants were instructed to remain motionless during the entire scanning procedure. Calibration and analysis were always performed by the same specialized laboratory technician. Equipment calibration followed the manufacturer's recommendations. The software generated standard lines that set apart the limbs from the trunk and head. These lines were adjusted by the same technician using specific anatomical points determined by the manufacturer. Regarding dominant femoral neck assessment, standard position was used with anterior-posterior scanning of the proximal femur.

#### 2.4.3 Physical function assessment

Before the physical function assessment, all participants completed a 10 min warm-up of low intensity walking with 5 min of stretching exercises for major muscle groups. The physical function parameters were evaluated by 30-sec chair‐stand test (repetitions/30-sec) for lower body strength [40]; SMBT for upper body strength (with a 3 kg medicine ball) [41]; TUG test for agility/dynamic balance [40]; and 6 MWT for aerobic capacity [40].

#### 2.4.4 Muscle strength

At the start of each single test, the participant was asked to relax in order for the passive effect of gravity on the limb be registered. The range of motion assessed was between 100° and 0° at full leg extension. Verbal encouragement was provided as stimulation for the subject to exert maximal effort. Subsequently, the maximal unilateral isokinetic strength for the dominant side in the knee extensors and flexors were measured during concentric actions at 60°/s with three repetitions using an isokinetic dynamometer (Biodex System 3, Biodex Corp., Shirley, NY, USA), according to the recommendations provided in the Biodex Isokinetic Manual and in the previous study [39].

Handgrip strength for the dominant side, recorded in kg, was measured by the handgrip strength test using a hydraulic handgrip dynamometer (JAMAR®, serial no. 2016100256). Before the assessment, the dynamometer was adjusted to each participant's hand. Participants were asked to stand with their feet shoulder-width apart, to look straight ahead, and with their elbows fully extended in a vertical position.

Thereafter, the participants were instructed to squeeze the grip with full force without bringing the arm close to the trunk. Two attempts were performed with a 1-min break, for at least 2 s [42]. The value registered was the average of two attempts.

### 2.5 Statistical analysis

Descriptive statistic was used to characterize the total sample, through means and SD for quantitative variables while frequencies and percentages for qualitative variables. All of them were checked for homoscedasticity by Levene tests, and for normality distribution by the Kolmogorov-Smirnov for total sample and by the Shapiro-Wilk for the groups divided by sex.

Semi partial correlations (Pearson product–moment correlation coefficient (*r*)) were used to verify the relationship between PhA and physical function, muscle strength and bone indicators controlling for age. When a variable did not assume a normal distribution, the Spearman correlation coefficient (*rho*) was used. The following magnitude of the correlations' effect sizes (ES) was considered for interpretation: <0.1, (trivial); 0.1–0.3, (small); >0.3–0.5, (moderate); >0.5–0.7, (large); >0.7–0.9, (very large); and >0.9, (virtually perfect) [43]. Comparisons between sexes for quantitative variables were analysed by independent sample *t*-test, while the chi-square test was used for qualitative variables. The Hopkins’ thresholds for ES statistics were used, as follows: ≤0.2, trivial; >0.2, small; >0.6, moderate, >1.2, large, >2.0, very large and >4.0, nearly perfect [44]. Finally, multiple regression analysis was performed to further assess if PhA can predict the dependent variables, after adjusting for potential confounders, namely, age, sex, lean mass, and BMI. All data were analysed using statistical software IBM SPSS for Windows version 26 (IBM Corp., Armonk, NY, USA).

## 3 Results

The general characteristics of the sample are summarized in Table 1. Men were significantly older and taller than women. In addition, PhA was higher in men.

**Fig. 2:**
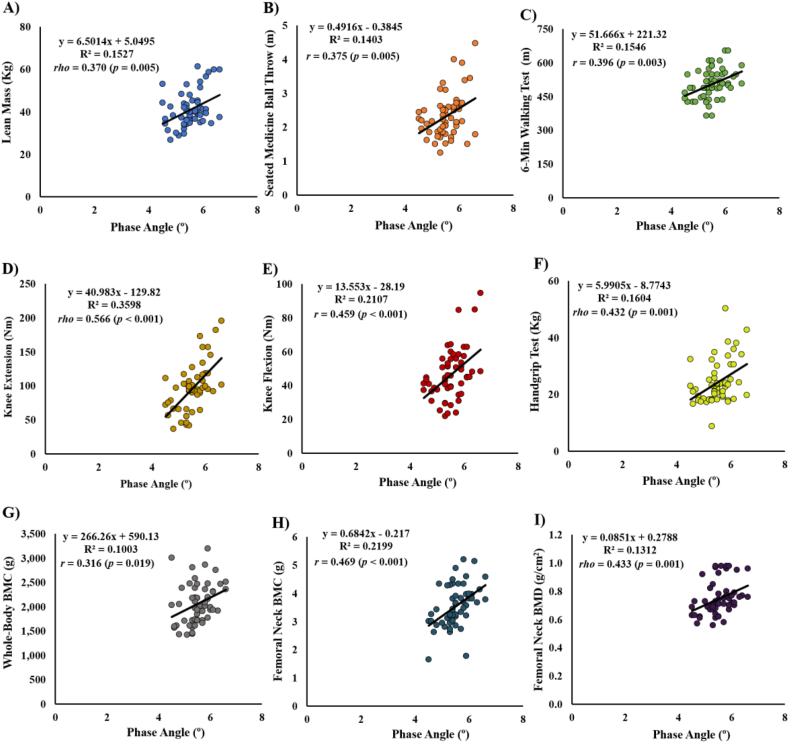
Correlations between phase angle and: **A),** lean mass; **B)**, seated medicine ball throw; **C),** 6-min walking test; **D)**, knee extension; **E)**, knee flexion; **F)**, handgrip test; **G)**, whole-body BMC; **H)**, femoral neck BMC; and **I)**, femoral neck BMD after controlling for age.

No correlations were found between PhA with FM, BMI, TUG test, and whole-body BMD for all participants (Table 2). Despite these results, PhA showed a significant correlation with weight, FM (%), lean mass (Fig. 2A), SMBT (Fig. 2B), 30s chair‐stand test, 6 MWT (Fig. 2C), knee extension (Fig. 2D), knee flexion (Fig. 2E), handgrip test (Fig. 2F), whole body BMC (Fig. 2G), femoral neck BMC (Fig. 2H) and BMD (Fig. 2I) after controlling for age.

**Table 2:** Pearson's correlation between phase angle with body composition, physical function, muscle strength and bone indicators after controlling for age by sex.

Measures	Men (N = 14)			Women (N = 42)			Total (N = 56)		
	Mean ± SD	Corr. Value	*p*-value	Mean ± SD	Corr. Value	*p*-value	Mean ± SD	Corr. Value	*p*-value
*Body Composition*									
Weight (kg)	75.69 ± 13.89	0.262	0.387	68.67 ± 10.48	0.147	0.358	70.43 ± 11.71	0.277	0.041^a^
Fat Mass (kg)	21.52 ± 5.79	0.378	0.203^c^	28.66 ± 5.69	0.073	0.648	26.87 ± 6.47	−0.041	0.764
Fat Mass (%)	32.86 ± 3.99	0.110	0.710^c^	41.41 ± 5.57	−0.100	0.532	38.39 ± 6.64	−0.333^d^	0.013^a^
Lean Mass (kg)	50.91 ± 8.31	0.171	0.577^c^	37.64 ± 5.05	0.181	0.256	40.96 ± 8.30	0.370^d^	0.005^a^
BMI (kg/m^2^)	27.30 ± 3.41	0.510	0.075	28.36 ± 4.65	0.102	0.527	28.09 ± 4.37	0.096	0.487
*Physical Function*									
30 -sec Chair‐Stand Test (rep)	18.64 ± 8.52	0.531	0.037^a^	16.88 ± 6.07	0.602^d^	0.030^a^	17.32 ± 6.72	0.314^d^	0.019^a^
Timed Up & Go Test (s)	6.06 ± 1.24	−0.605	0.029^a^	6.50 ± 1.23	0.016^d^	0.919	6.39 ± 1.24	−0.146^d^	0.287
Seated Medicine Ball throw (m)	3.03 ± 0.75	0.520	0.068^c^	2.09 ± 0.42	−0.025	0.877	2.33 ± 0.65	0.375	0.005^a^
6-Min Walking Test (m)	526.55 ± 82.39	0.669	0.012^a^	500.07 ± 58.61	0.292	0.064	506.68 ± 65.57	0.396	0.003^a^
*Muscle Strength*									
Knee extension 60° (Nm)	135.39 ± 33.07	0.682	0.010^a^^c^	83.58 ± 22.92	0.418^d^	0.007^a^	96.53 ± 34.09	0.566^d^	<0.001^b^
Knee flexion 60° (Nm)	59.28 ± 17.96	0.522	0.067^c^	42.46 ± 10.80	0.209	0.189	46.66 ± 14.74	0.459	<0.001^b^
Handgrip Test (kg)	33.91 ± 7.87	0.121	0.694^c^	21.11 ± 3.64	0.322^d^	0.040^a^	24.31 ± 7.46	0.432^d^	0.001^b^
*Bone indicators*									
Whole body BMC (g)	2464.14 ± 434.03	−0.533	0.061^c^	1926.25 ± 320.05	0.407	0.008^a^	2060.72 ± 419.67	0.316	0.019^a^
Whole body BMD (g/cm^2^)	1.14 ± 0.11	−0.489	0.090	1.06 ± 0.18	0.365^d^	0.019^a^	1.08 ± 0.17	0.128	0.352
Femoral neck BMC (g)	3.84 ± 1.04	0.051^d^	0.868	3.47 ± 0.58	0.490^d^	0.001^b^	3.56 ± 0.73	0.469	<0.001^b^
Femoral neck BMD (g/cm^2^)	0.78 ± 0.10	0.058^d^	0.852	0.74 ± 0.12	0.460^d^	0.003^a^	0.75 ± 0.12	0.433^d^	0.001^b^

Abbreviations: Kg, kilograms; %, percent; BMI, body mass index; rep, repetitions; s, seconds; m, meters; Nm, newton-meter; BMC, bone mineral content; BMD, bone mineral density; g, grams; cm; centimeters; SD, standard deviation; Corr, correlation.

a Differences for *p* ≤ 0.05.

b Differences for *p* ≤ 0.001.

c Differences between males and females (*p* ≤ 0.05).

d Partial correlation performed by Spearman correlation test.

For men and women, PhA presented a significant correlation with 30s chair‐stand test and knee extension. On other hand, TUG test and 6 MWT were only correlated with PhA for men, and handgrip test, whole body BMC and BMD and femoral neck BMC and BMD were only correlated with PhA for women.

Lastly, there were significant differences between men vs. women for FM in kg and percent (p < 0.001; ES = −1.23 [−1.89; −0.59], large ES) and (p < 0.001; ES = −4.19 [−5.24; −3.24]), large ES), respectively, for lean mass (p < 0.001; ES = 2.18 [1.47; 2.94]), large ES), for SMBT (p < 0.001; ES = 1.79 [1.11; 2.49]), large ES), knee extension (p < 0.001; ES = 1.98 [1.29; 2.72]), large ES), knee flexion (p < 0.001; ES = 1.28 [0.64; 1.95]), large ES), handgrip test (p < 0.001; ES = 2.53 [1.78; 3.33]), large ES) and whole body BMC (p < 0.001; ES = 1.51 [0.86; 2.20]), large ES).

Fig. 2 illustrates the relation between PhA and the performance in some physical functional tests, as well as muscle strength and bone indicators.

Table 3, Table 4, Table 5 display the multiple regression models for the physical function, muscle strength and bone indicators, respectively, including adjustments for age, sex, lean mass, and BMI (Model 4). For physical function, SMBT (β = 2.449; *p* < 0.001; R^2^ = 0.629) and 6 MWT (β = 80.578; *p* = 0.004; R^2^ = 0.214) remained positively significant with PhA.

**Table 3:** Multiple regression analysis between phase angle and physical function.

Physical Function	β (CI 95%)	R	Adjusted R^2^	*p-value*
30-sec *Chair‐Stand Test*				
Phase Angle	−3.918 (−23.451 to 15.615)	0.285	0.064	0.033^a^
Model 1	−4.302 (−48.797 to 40.194)	0.285	0.047	0.105
Model 2	−2.534 (−58.562 to 53.495)	0.286	0.029	0.215
Model 3	−22.099 (−83.807 to 39.609)	0.344	0.049	0.163
Model 4	−18.924 (−86.534 to 48.686)	0.345	0.031	0.258
*Timed Up &Go Test*				
Phase Angle	9.175 (5.495–12.855)	0.203	0.023	0.134
Model 1	9.734 (1.353–18.115)	0.204	0.005	0.325
Model 2	7.654 (−2.856 to 18.164)	0.223	−0.005	0.445
Model 3	4.952 (−6.733 to 16.636)	0.265	−0.003	0.437
Model 4	4.319 (−8.482 to 17.121)	0.267	−0.022	0.577
*Seated Medicine Ball Throw*				
Phase Angle	−0.383 (−2.224 to 1.458)	0.374	0.124	0.004^a^
Model 1	0.150 (−4.041 to 4.340)	0.376	0.109	0.018^a^
Model 2	7.638 (3.679–11.597)	0.719	0.489	0.001^b^
Model 3	4.435 (0.488–8.383)	0.787	0.590	0.001^b^
Model 4	2.449 (−1.627 to 6.525)	0.814	0.629	0.001^b^
*6-Min Walking Test*				
Phase Angle	221.322 (38.564–404.081)	0.393	0.139	0.003^a^
Model 1	−24.779 (−434.166 to 384.609)	0.427	0.152	0.005^a^
Model 2	−78.647 (−593.589 to 436.294)	0.430	0.137	0.013^a^
Model 3	209.041 (−339.500 to 757.581)	0.517	0.210	0.003^a^
Model 4	80.578 (−513.379 to 674.535)	0.534	0.214	0.004^a^

Note: Model 1 phase angle adjusted for age; Model 2, phase angle adjusted for age and sex; Model 3: phase angle adjusted for age, sex, and lean mass; Model 4: phase angle adjusted for age, sex, lean mass, and body mass index.

a Differences for *p* ≤ 0.05.

b Differences for *p* ≤ 0.001.

**Table 4:** Multiple regression analysis between phase angle and muscle strength.

Muscle Strength	β (CI 95%)	R	Adjusted R^2^	*p-value*
*Knee extension 60°*				
Phase Angle	−129.822 (−212.524 to −47.121)	0.600	0.348	<0.001^b^
Model 1	−143.180 (−331.526 to 45.166)	0.600	0.336	<0.001^b^
Model 2	197.426 (21.141–373.712)	0.804	0.626	<0.001^b^
Model 3	55.560 (−120.435 to 231.556)	0.849	0.699	<0.001^b^
Model 4	26.725 (−165.059 to 218.508)	0.851	0.697	<0.001^b^
*Knee flexion 60°*				
Phase Angle	−28.190 (−67.882 to 11.502)	0.459	0.196	<0.001^b^
Model 1	−34.231 (−124.629 to 56.168)	0.459	0.181	0.002^a^
Model 2	74.566 (−27.367 to 176.500)	0.606	0.331	<0.001^b^
Model 3	31.775 (−79.452 to 143.003)	0.635	0.357	<0.001^b^
Model 4	10.227 (−110.685 to 131.139)	0.643	0.355	<0.001^b^
*Handgrip Test*				
Phase Angle	−8.774 (−29.509 to 11.960)	0.401	0.145	0.002^a^
Model 1	−22.618 (−69.658 to 24.422)	0.409	0.136	0.008^a^
Model 2	73.450 (34.644–112.257)	0.802	0.622	<0.001^b^
Model 3	34.147 (−1.443 to 69.737)	0.873	0.743	<0.001^b^
Model 4	14.477 (−21.784 to 50.739)	0.891	0.774	<0.001^b^

Note: Model 1, phase angle adjusted for age; Model 2, phase angle adjusted for age and sex; Model 3: phase angle adjusted for age, sex, and lean mass; Model 4: phase angle adjusted for age, sex, lean mass, and body mass index.

a Differences for p ≤ 0.05.

b Differences for p ≤ 0.001.

**Table 5:** Multiple regression analysis between phase angle and bone indicators.

Bone indicators	β (CI 95%)	R	Adjusted R^2^	*p*-value
*Whole body BMC*				
Phase Angle	590.132 (−616.575 to 1796.840)	0.317	0.084	0.017^a^
Model 1	−427.625 (−3158.601 to 2303.352)	0.335	0.078	0.043^a^
Model 2	3363.465 (412.478–6314.451)	0.588	0.308	0.001^b^
Model 3	528.462 (−2245.209 to 3302.133)	0.737	0.507	0.001^b^
Model 4	−426.977 (−3386.145 to 2532.190)	0.753	0.524	0.001^b^
*Whole body BMD*				
Phase Angle	0.843 (0.345–1.341)	0.129	−0.002	0.342
Model 1	0.584 (−0.547 to 1.716)	0.147	−0.015	0.562
Model 2	1.086–0.320 to 2.491)	0.219	−0.015	0.462
Model 3	0.470 (−1.059 to 1.999)	0.328	0.037	0.207
Model 4	0.228 (−1.439 to 1.895)	0.343	0.029	0.267
*Femoral neck BMC*				
Phase Angle	−0.218 (−2.167 to 1.731)	0.469	0.206	0.001^b^
Model 1	−1.568 (−5.988 to 2.852)	0.476	0.198	0.001^b^
Model 2	−1.192 (−6.756 to 4.372)	0.477	0.183	0.004^a^
Model 3	−3.431 (−9.517 to 2.655)	0.518	0.211	0.003^a^
Model 4	−5.781 (−1.801 to −12.227)	0.563	0.249	0.001^b^
*Femoral neck BMD*				
Phase Angle	0.273 (−0.060 to 0.605)	0.365	0.117	0.006^a^
Model 1	0.144 (−0.613 to 0.901)	0.368	0.103	0.021^a^
Model 2	0.131 (−0.822 to 1.084)	0.368	0.086	0.054
Model 3	−0.324 (−1.354 to 0.707)	0.447	0.137	0.021^a^
Model 4	0.622 (−1.730 to 0.486)	0.479	0.153	0.020^a^

Note: Model 1, phase angle adjusted for age; Model 2, phase angle adjusted for age and sex; Model 3: phase angle adjusted for age, sex, and lean mass; Model 4: phase angle adjusted for age, sex, lean mass, and body mass index.

Abbreviations: BMC, bone mineral content; BMD, bone mineral density.

a Differences for *p* ≤ 0.05.

b Differences for *p* ≤ 0.001.

Regarding muscle strength, the results revealed that PhA exhibited a significant positive association with knee extension (β = 26.725; *p* < 0.01; R^2^ = 0.697), knee flexion (β = 10.227; *p* < 0.01; R^2^ = 0.355), and handgrip (β = 14.477; *p* < 0.01; R^2^ = 0.774).

Lastly, for bone indicators, even after controlling for potential confounders (model 4), PhA showed a significant relation with whole-body BMC (β = −426.977; *p* < 0.01; R^2^ = 0.524), femoral neck BMC (β = −5.781; *p* = 0.001; R^2^ = 0.249), and femoral neck BMD (β = 0.622; *p* = 0.020; R^2^ = 0.153).

## 4 Discussion

The aim of the cross-sectional exploratory pilot study was twofold: a) to verify the potential relationship between PhA with body composition parameters, physical function, muscle strength, and bone indicators, and b) to analyze the ability of PhA to predict physical function, muscle strength and bone indicators after adjusting for potential confounders in independent older adults. Accordingly, the key findings were that PhA was significantly associated with physical function, especially SMBT, and 6 MWT; muscle strength; and bone indicators, namely femoral neck BMC. The novelty of this study was the ability of PhA to predict muscle strength, and some outcomes of physical function and bone indicators in older adults. In this sense, the study hypothesis was confirmed since PhA can predict, and it is associated with, muscle strength and some factors related to physical function and bone quality. Below, these results are discussed in more detail considering each parameter.

### 4.1 Body composition

The present study revealed a PhA mean of 5.52° for the total sample, 5.83° for men, and 5.42° for women (Table 1). The observed significant difference between sexes is in line with previous studies in older adults [15,16]. Additionally, this study presented higher values than those reported in other studies performed in older adults with a higher mean age [18,27,45]. A study conducted by Gonzalez et al. [46] with 1442 participants (843 women and 599 men) between 31 and 61 years old from different races/ethnicity concluded that the age is the most important biological determinant of PhA variation, therefore, age was also considered as the most significant PhA predictor in men and women. This was also confirmed in the present study considering the SMBT, 6 MWT, all muscle strength variables, whole body BMC, femoral neck BMC and BMD.

Aging is a process that involves several physiological impairments [47]. Notably, the loss of muscle mass, accompanied by an increase in the extracellular fluids leads to a decrease in PhA values [28,46]. Norman et al. [14] also concluded that the PhA decreases when age increases due to the increasing FM in advanced age. The present study revealed moderate associations for the total sample between PhA with %FM and lean mass (Fig. 2A). In this regard, Kilic et al. [19] reported an association between PhA with muscle mass in 263 older adults. Additionally, Basile et al. [27] reported a strong association between PhA with muscle mass (*p* < 0.001; *r* = 0.600) in 207 older adults (mean age, 76.2 ± 6.7 years) admitted for multidimensional geriatric evaluation. These authors concluded that the PhA is linearly related to muscle mass, irrespective of several factors such as age. The authors consider PhA an inexpensive marker to preventively detect sarcopenia in older adults in whom a moderately low skeletal muscle is associated with shorter survival rates which is also reinforced by the results of the present study.

### 4.2 Physical function

Higher values of PhA were correlated with better physical function, except for the TUG test. These results are in line with previous studies in this field [16,25,28]. Nevertheless, the present study revealed new insights regarding physical function, particularly with the association between PhA and SMBT (Fig. 2B). This test is related to the muscle power and explosive force [41]. Consequently, this association may represent that better cellular and membrane integrity enhances the ability to recruit motor units and improves the capacity of muscle activation and force production [48]. In a study performed with breast cancer patients, Martins, Oliveira et al. [49] showed that the group with higher PhA values performed better in the SMBT. However, the specificities of the population under that study limit the extrapolation of the results to independent older adults. Nonetheless, the inclusion of this test is recommended in future studies due to the fact to be highly reliable and reasonably valid test for assessing upper body muscular power in the older adult [41].

Regarding the 6 MWT, it was also observed a positive association with the PhA (Fig. 2C). This finding is in line with previous research. For instance, Mullie et al. [23] conducted a study with 277 subjects and reported a moderate association between the 6 MWT and PhA and revealed that low PhA values before heart surgery were associated with low walking speed. In the same sense, a recent study by Germano et al. [15] showed an association between the PhA and walking speed through the 4-m walking speed test. Thus, the 6 MWT results in the current study corroborated that older adults with high values of PhA could have better cardiorespiratory capacity.

When lower limbs were assessed through the 30-sec chair‐stand test, it was found a small association between this test and PhA. This result is in accordance with a previous study conducted by Matias et al. [16] that exposed a small association between this test and PhA. Jones et al. [50] referred that it is fundamental to measure lower body strength to assess the physical function in older adults and recommended the use of the 30-sec chair-stand test. From the fact that lower levels of body strength are an important factor in the loss of functionality with aging, the association found in the present study suggests that the PhA could be a good indicator for both muscle quality and quantity, and it is a sensitive marker for physical function [18].

Finally, after controlling for potential confounders, the present results confirmed the outcomes of previous studies that suggested that PhA is a predictor of physical function [15,16,23,27,28]. Interestingly, several authors have stated that PhA provides complementary information about physical function, such as the level of cell integrity and the cellular function [14,51]. It is important to highlight that PhA can explain 63% (Model 4) of the result of the SMBT. The promising results could be explained by the age of the participants under study (68.29 ± 3.01 years) and their characteristics (independent older adults).

A recent study performed by Bellido et al. [12] encouraged the researchers and clinicians to evaluate whether PhA has a good predictive capacity in relation to the diagnostic components of sarcopenia (physical performance, muscle mass, and strength). In this sense, taking into account the available literature [15,18,25,28,51], despite the fact that the present study is an experimental cross-sectional study, the results demonstrated an increased ability of PhA to predict physical function and provide new directions for future research.

### 4.3 Muscle strength

A large association between PhA and knee extension (Fig. 2D) and moderate associations between PhA and knee flexion (Fig. 2E) as well as the handgrip test (Fig. 2F) were found. In addition, the linear regression analysis results showed higher values for adjusted R^2^ for each outcome (0.699 for knee extension, [Model 3]; 0.357 for knee flexion, [Model 3]; and 0.774 for handgrip test, [Model 4]). The results suggested that high values of PhA may reflect a good neuromuscular system. A possible explanation for the observed results could be that the PhA represents the quantity and the integrity of cells with their respective cell membranes [52,53]. Interestingly, a recent study about the future lines of research on PhA stated that this marker is a raw parameter of cellular health, equivalent to “*the electrocellgram®*” [12]. The results of the present study corroborated this statement. The results can also show the relationship of PhA with cell permeability as well as the amounts of extracellular and intracellular fluids [54] and can express the quality of soft tissue [14,53]. These scenarios may assist in the increase of neural drive via corticospinal pathways, increased motor neuron and/or muscle fiber excitability, the number of active motor units, and/or conduction velocity [55]. However, Yamada et al. [18] assumed that the relationship between PhA and muscle strength is not completely understood. Hence, these results provide a solid basis for understanding those relationships and encourage authors to include these outcomes in future studies.

On the one hand, few studies have considered the relationship between PhA with isokinetic strength parameters [14,56], however the results in these studies were obtained through the Barthel Index of the activities of daily living. On the other hand, several studies have assessed the muscle strength through the handgrip test [15,18,24,27,57,58]. The prediction model with handgrip test showed higher values than the model used in the Germano et al. [15] study (0.774 *vs.* 0.321, respectively). Additionally, the values were also higher when compared with the model showed in a study performed by Basile et al. [27] (0.774 *vs.* 0.319, respectively). The results of the present study confirmed previous studies and provided more details regarding the relationship between muscle strength and PhA.

### 4.4 Bone indicators

To the authors knowledge, only one study investigated the relationship between PhA and BMD in older adults [22]. Although the study conducted by Antunes et al. [22] had included the BMD values for whole-body, femur, neck, and forearm regions, it did not include the BMC values. The relationship between PhA and bone indicators is still not completely understood. Therefore, the current findings provide more details regarding this relationship. It was observed that PhA is moderate related to whole-body BMC (Fig. 2G), femoral neck BMC (Fig. 2H) and BMD (Fig. 2I). Interestingly, in a previous study the whole-body BMD showed a positive correlation with PhA, but in the present study this association did not occur. Regarding the femoral neck BMD, the present study confirmed the previous result of a positive correlation with PhA [22].

Even though it is not possible to establish a causal relationship, it seems that these associations could be related to the fact that it is well established that there is a relationship between muscle mass and bone density [59]. The causes of bone mass loss are multifactorial and similar to the causes of muscle mass loss [60]. Beyond aging, the studies highlighted the poor blond flow to the muscles, mitochondrial dysfunction, an increase in pro-inflammatory cytokines and hormonal changes, especially in women after menopause [61]. In addition, Karasik & Cohen-Zinder [62] referred that the Alpha Actinin-3, that regulates the muscular power performance, is also associated with the bone mass loss. On the other hand, on a biomechanical level, the muscle force affects bone strength and density [63]. Additionally, Douchi et al. [64] added that this force is influenced by how much body mass the muscles and bones support, which can promote positive changes in BMD and BMC.

Finally, PhA may be related with bone indicators, particularly femoral neck BMC and BMD. The present findings revealed the PhA can explain approximately 52% of whole-body BMC, 25% and 15% of femoral neck BMC and BMD, respectively. Consequently, the results may suggest that higher cellularity, cell membrane integrity and better cell function, expressed by PhA, is related with good levels of BMD and BMC, which can indicate that PhA can be used as a marker of bone quality.

### 4.5 Limitations

Limitations of our pilot study are mainly related to the cross-sectional design. In this regard, it was not possible to demonstrate a clear cause/effect relationship due to the relatively modest sample size. Nevertheless, the present study considered the sample size calculation. Secondly, the measurement differences between BIA devices from different manufacturers, compromises the extrapolation of these findings to single-frequency or other multi-frequency BIA instruments [65]. Harmonization of technology, as well as crosscalibration of electrical resistors is needed to facilitate direct comparison of results from different studies as well as the application of generally accepted reference values [14,65]. Despite the promising results, the use of the BIA is also subject to limitations as the physiological and pathological conditions that could influence the measurement. Finally, no physical activity questionnaire was applied, which may be a limiting factor, as the PhA could also be related to the amount of physical activity, and it was not contemplated in quantitative terms.

Despite these limitations, this pilot research anticipates forthcoming, larger studies in order to determine the ability of PhA to predict the several outcomes, related with physical function, muscle strength and bone indicators in community-dwelling independent older adults.

## 5 Conclusion

The results of the preliminary analysis of the relationship between PhA and body composition, muscle strength and some factors related to physical function suggested that PhA is linked to muscle strength and some factors related to physical function and bone quality. Thus, the main novelty of this study is that PhA can be considered a potential predictor of physical function and muscle strength in community-dwelling older adults. In addition, the results regarding bone indicators imply that PhA can be used as a marker of bone quality. Finally, the present study's results reinforce the importance of using the gold standard techniques and tools (InBody®, Dexa®, Biodex®, and Jamar®) in future research.

## Authorship contribution statement

**Alexandre Duarte Martins:** Conceptualization, Methodology, Formal analysis, Investigation, Resources, Data curation, Writing – original draft, Visualization, Project administration, Funding acquisition.

**João Paulo Brito:** Conceptualization, Methodology, Writing – review & editing, Visualization, , Supervision.

**Nuno Batalha:** Writing – review & editing, Visualization, Supervision.

**Rafael Oliveira:** Writing – review & editing, Visualization, Funding acquisition.

**Jose A. Parraca:** Writing – review & editing and Visualization.

**Orlando Fernandes:** Writing – review & editing, Visualization, Funding acquisition, Supervision.

All authors approved the final version of the manuscript.

## Funding

This research was funded by the 10.13039/501100001871Fundação para a Ciência e a Tecnologia, I.P., Grant/Award Numbers 2021.04598.BD; UIDP/04923/2020 and UIDP/04748/2020. The funders had no role in the design of the study; in the collection, analyses or interpretation of data; in the writing of the manuscript or in the decision to publish the results.

## Data availability statement

Data will be made available on request.

## Additional information

Supplementary content related to this article has been publish online at [URL].

## Declaration of competing interest

The authors declare that they have no known competing financial interests or personal relationships that could have appeared to influence the work reported in this paper.
